# Association between time-varying weighted hemoglobin and all-cause mortality in patients with acute myocardial infarction-related cardiogenic shock

**DOI:** 10.3389/fcvm.2025.1516100

**Published:** 2025-05-14

**Authors:** Xia Liu, Tianbo Gong, Yongpeng Zhang

**Affiliations:** Department of Emergency Medicine, Dongying City People’s Hospital, Dongying, Shandong, China

**Keywords:** hemoglobin, acute myocardial infarction, cardiogenic shock, all-cause mortality, anemia

## Abstract

**Background and aims:**

Anemia has been implicated in prognosis across ischemic heart diseases. This study aimed to investigate the association between time-weighted average hemoglobin (TWA-Hb) and all-cause mortality in patients with acute myocardial infarction-related cardiogenic shock (AMI-CS).

**Methods and results:**

We conducted a retrospective analysis of 765 patients diagnosed with AMI-CS using data from the MIMIC-IV database (2008–2019). Kaplan–Meier survival analysis demonstrated that lower TWA-Hb levels were associated with higher cumulative mortality rates at 28 days, 90 days, 6 months, and 1 year (log-rank *P* = 0.002, 0.006, 0.048, and 0.005, respectively). Landmark analyses further revealed a sustained increase in mortality risk associated with lower TWA-Hb during the 28-day to 1-year follow-up period. Multivariable Cox regression analysis identified low TWA-Hb as an independent predictor of mortality risk at 90 days (*P* = 0.026), 6 months (*P* = 0.023), and 1 year (*P* = 0.021). Each one-unit increase in TWA-Hb was associated with a 0.93-, 0.76- and 0.71-fold decrease in the risk of 90-day, 6-month, and 1-year mortality, correspondingly. Subgroup analyses stratified by age, BMI, and comorbidities consistently supported these findings (all *P* < 0.05).

**Conclusion:**

Low TWA-Hb is associated with long-term mortality in patients with AMI-CS. These findings imply that the application of this indicator in clinical practice could improve long-term risk stratification.

## Introduction

Cardiogenic shock (CS), characterized by critically reduced cardiac output leading to systemic circulatory failure, multi-organ hypoperfusion, and hypoxia, carries a mortality rate exceeding 40% despite therapeutic advances (1). As a complication of 3%–13% of acute myocardial infarction (AMI) cases, acute myocardial infarction-related cardiogenic shock (AMI-CS) remains one of the most predominant etiologies associated with high mortality (2–4). Current guidelines emphasize coronary revascularization, vasopressors, and mechanical circulatory support (MCS) to restore tissue oxygenation in AMI-CS patients (5). However, only immediate culprit vessel revascularization and Impella device deployment have demonstrated consistent survival benefits (2, 6, 7), with mortality persisting to be substantial (8, 9).

Hemoglobin (Hb), the principal oxygen-carrying component of erythrocytes, plays a pivotal role in maintaining systemic oxygen delivery–demand equilibrium (10). While anemia has been independently associated with worsened outcomes in both general CS populations and AMI patients without shock (11–14), its prognostic significance in AMI-CS remains underexplored. Existing studies are limited by reliance on single-point Hb measurements (15), neglecting the dynamic nature of Hb fluctuations during hospitalization and their cumulative impact on clinical outcomes.

To address this gap, our study investigated the association between time-weighted average hemoglobin (TWA-Hb)—a novel metric quantifying cumulative Hb exposure—and all-cause mortality in AMI-CS patients. This approach enables comprehensive evaluation of Hb trajectory's prognostic value beyond conventional static assessments.

## Methods

### Study population

The current study is a retrospective study based on the Medical Information Mart for Intensive Care IV (MIMIC-IV) database, a common and single-center database that was developed by the Computational Physiology Laboratory of Massachusetts Institute of Technology. This database comprises comprehensive and high-quality medical information regarding individuals admitted to the intensive critical care units of Beth Israel Deaconess Medical Centre between 2008 and 2019.

**Figure 1:**
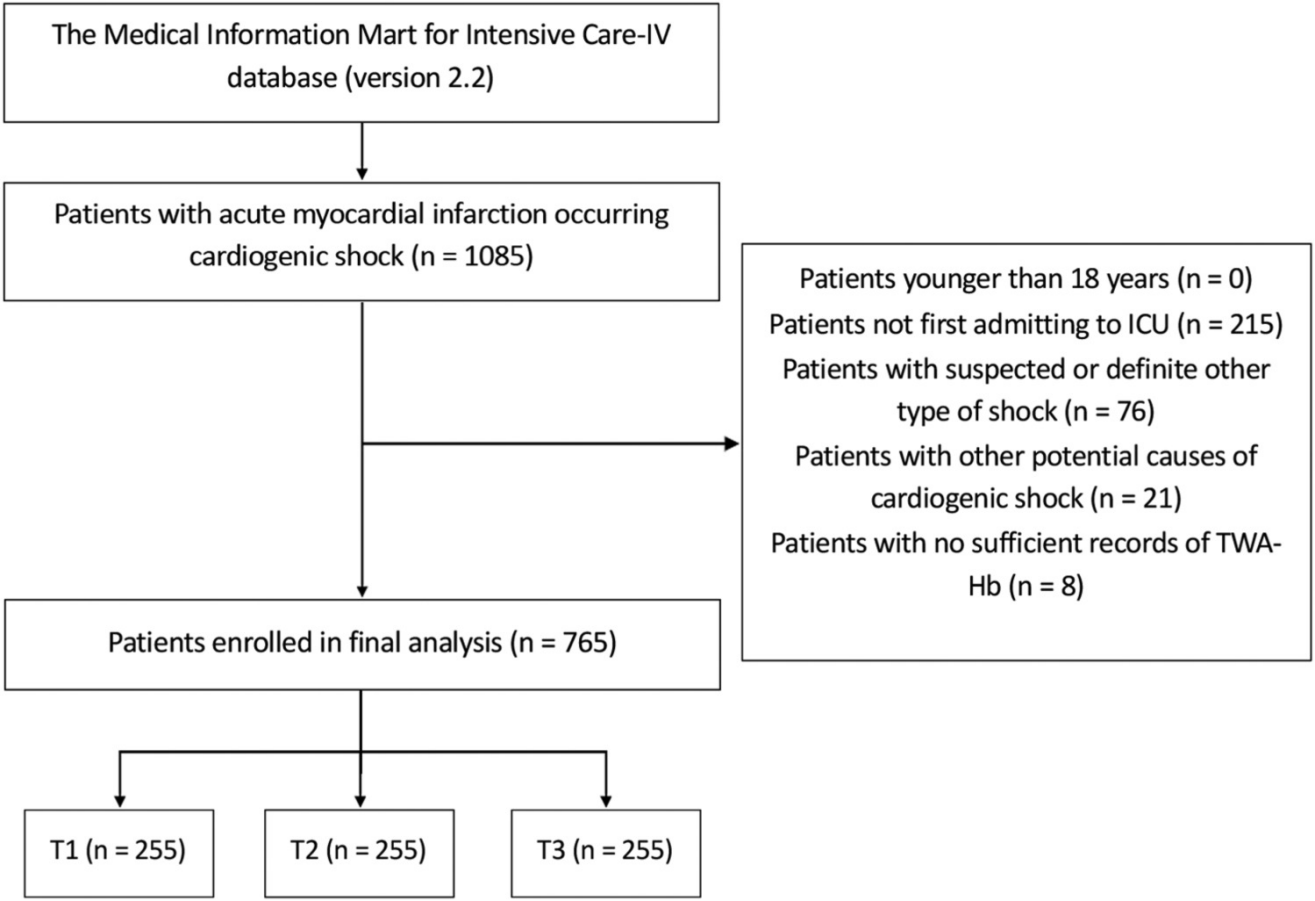
Flow diagram of patient selection. T, tertile; ICU, intensive care unit; TWA-Hb, time-varying weighted hemoglobin.

Patients diagnosed with AMI occurring CS were enrolled in this analysis using the International Classification of Diseases, 9th and 10th Revision; thereinto, only the first in-hospital records were analyzed for individuals with multiple hospital admissions. Patients were excluded if younger than 18 years; if with other types of shock, such as septic shock, allergic shock, neurogenic shock, hemorrhagic shock, or shock with unknown origin; if with other potential causes of CS, such as acute myocarditis and cardiac tamponade; or if without sufficient records of TWA-Hb during hospitalization. Finally, a total of 765 patients were recruited and further separated into three groups in accordance with the tertiles of TWA-Hb. The patient screening flowchart is presented in Figure 1.

### Data extraction

Variables extracted included as follows: (1) demographics and vital signs at admission, including age, sex, weight, height, ethnicity, temperature, respiratory rate, heart rate, systolic blood pressure, and diastolic blood pressure; (2) comorbidities, including smoking, hypertension, diabetes mellitus (DM), chronic heart failure (CHF), atrial fibrillation, cerebrovascular disease (CVD), chronic obstructive pulmonary disease, peripheral arterial disease, chronic kidney disease (CKD), rheumatic disease, cancer, and severe liver disease; (3) baseline laboratory findings, including pH, PaCO_2_, PaO_2_, SpO_2_, white blood cell (WBC), red blood cell (RBC), platelet, potassium, sodium, chloride, bicarbonate, creatine kinase-myocardial band (CK-MB), and lactate; (4) severity of illness scores at admission, including Simplified Acute Physiology Score II (SAPSII), Logistic Organ Dysfunction System (LODS), Oxford Acute Severity of Illness Score (OASIS), Charlson Comorbidity Index (CCI), and Sepsis-Related Organ Failure Assessment (SOFA) score; (5) in-hospital treatments, including mechanical ventilation (MV), intra-aortic balloon pump (IABP), renal replacement therapy (RRT), vasoactive agents, RBC input, and plasma input. Every Hb value and corresponding testing time during hospitalization was recorded, and the TWA-Hb was calculated as the time-weighted sum of the Hb values obtained at each time as: {Hb_time1_*(time2 − time1) + Hb_time2_*(time3 − time2) + Hb_time3_*(time4 − time3) + … + Hb_time (*n* − 1)_*[time (*n*) − time (*n* − 1)]} / (*n* − 1), where the Hb_time1_, Hb_time2_, Hb_time3_ are the Hb examined at the first, second, and third time after admission, respectively, and (time2 − time1), (time3 − time2), and (time4 − time3) are the specific time intervals in hours between consecutive examinations, and so forth.

### Clinical outcomes

The primary outcome was all-cause mortality at 1 year. The secondary outcomes were all-cause 28-day, 90-day, and 6-month mortality and the length of stay in hospital and ICU.

### Statistical analysis

Statistical analyses were conducted using SPSS (version 23.0, USA), R software (version 3.5.1, Austria), and GraphPad Prism (version 9.0, USA). Continuous variables were presented as mean ± standard deviation (SD) or median (interquartile range) and assessed by unpaired *t*-test ANOVA or the Mann–Whitney *U*/Kruskal–Wallis test based on the normality of values. Categorical variables were presented as absolute number (percentages) and evaluated by the chi-square test. Statistical significance was considered when two-tailed *P* < 0.05. The Kaplan–Meier survival analyses were performed to evaluate cumulative incidences of all-cause mortality and analyzed through a log-rank test. Landmark analysis was further applied to verify the predictive value of TWA-Hb for short-term (<28 days) and long-term (28 days to 1 year) mortality. Potential confounders were set in accordance with the results of univariate logistic regression analysis and clinical significance (Supplementary Table 1). The TWA-Hb was calculated in two patterns: (1) categorical variables and (2) continuous variables. For Cox regression analysis, three models were performed to evaluate the predictive value of TWA-Hb for all-cause mortality: (1) Model 1, unadjusted; (2) Model 2, adjusted for age and gender; (3) Model 3, adjusted for age, gender, BMI, ethnicity, temperature, respiratory rate, mean arterial pressure (MAP), hypertension, DM, CHF, CKD, cancer, pH, SpO_2_, RBC, bicarbonate, lactate, IABP use, MV use, RRT, vasoactive agents use, and RBC input. Cox regression analyses yielded hazard ratios (HR) along with 95% confidence intervals (CI) to present the results. Subgroup analyses were further performed utilizing Model 3 based on gender, age (>65 years and ≤65 years), BMI (>28.0 kg/m^2^ and ≤28.0 kg/m^2^), hypertension, DM, and CKD to explore the consistency of TWA-Hb's prognostic value for all-cause mortality.

## Results

After screening the data of 1,085 patients with AMI-CS, a total of 765 adult individuals were finally recruited in the present study, with a median age of 72.01 years (IQR: 71.11–72.90) and male patients accounting for 60.9% (*n* = 466). Of the study cohort, the 28-day, 90-day, 6-month, and 1-year mortality were recorded at 44.6%, 47.5%, 49.9%, and 53.2% of the populations, respectively, and the Hb level of the overall cohort was 10.33 g/dl (IQR: 10.21–10.46). The flow diagram of patient selection is shown in Figure 1.

### Baseline characteristics of the study population

The total population was stratified into three groups according to the TWA-Hb tertiles (T1, 6.94–9.33; T2, 9.33–10.94; T3, 10.94–18.07), and the baseline characteristics of enrolled individuals are shown in Table 1. The mean TWA-Hb value of each tertile was 8.6 g/dl (IQR: 8.2–9.0), 10.0 g/dl (IQR: 9.7–10.5), and 12.1 g/dl (IQR: 11.4–13.1), respectively. Populations in the highest tertile of TWA-Hb were younger; tended to be male; were exposed to higher levels of WBC, RBC, and CK-MB; and had lower severity of illness scores (SAPS II, LODS, CCI) on admission in comparison with the lower group. In those with the highest TWA-Hb, fewer participants had DM, CKD, and rheumatic disease and were treated with RRT and vasoactive agents; however, more individuals had concomitant hypertension. Meanwhile, larger amounts of RBC were input in the T3 group. Moreover, individuals in the highest tertile of TWA-Hb had lower LOS-ICU (4.7 vs. 4.0 vs. 3.2 days; *P* < 0.0001) and LOS-H (12.7 vs. 9.9 vs. 6.5 days; *P* < 0.0001), as well as lower 28-day (50.6 vs. 44.3 vs. 38.8%; *P* = 0.028), 90-day (53.3 vs. 47.5 vs. 41.6%; *P* = 0.029), 6-month (56.5 vs. 50.2 vs. 43.1%; *P* = 0.009), and 1-year (60.0 vs. 52.9 vs. 46.7%; *P* = 0.011) mortality compared with the lowest tertile (Table 1).

**Table 1:** Baseline characteristics of the study population.

Variables	Overall (*n* = 765)	T1 (*n* = 255)	T2 (*n* = 255)	T3 (*n* = 255)	*P*-value
Hb, g/L	10.3 (10.2–10.5)	8.6 (8.2–9.0)	(9.7–10.5)	12.1 (112.1 (11.4–13.1)	0.000
Age, years	72.0 (71.1–72.9)	73.5 (64.6–80.7)	73.9 (66.0–83.2)	70.2 (62.0–79.2)	0.010
Sex, *n* (%)					0.001
Male	466 (60.9)	134 (52.5)	158 (62.0)	174 (68.2)	
Female	299 (39.1)	121 (47.5)	97 (38.0)	81 (31.8)	
BMI, kg/m^2^	28.3 (27.7–28.9)	28.1 (23.8–32.2)	26.9 (23.7–31.7)	27.1 (24.1–31.5)	0.880
Ethnicity, *n* (%)					0.727
White	490 (64.1)	164 (64.3)	162 (63.5)	164 (64.3)	
Black	49 (6.4)	18 (7.1)	19 (7.5)	12 (4.7)	
Others	226 (29.5)	73 (28.6)	74 (29.0)	79 (31.0)	
Severity of illness					
SAPSII	46.3 (45.2–47.4)	47.0 (37.0–56.0)	45.0 (36.0–56.0)	41.0 (32.0–54.0)	0.004
LODS	6.7 (6.5–7.0)	7.0 (5.0–9.0)	7.0 (5.0–9.0)	6.0 (3.0–9.0)	0.001
OASIS	35.5 (34.8–36.2)	36.0 (30.0–42.0)	36.0 (30.0–43.0)	34.0 (26.0–41.0)	0.062
CCI	6.9 (6.7–7.1)	8.0 (6.0–9.0)	7.0 (5.0–8.0)	6.0 (4.0–8.0)	0.000
SOFA	2.8 (2.6–2.9)	2.0 (1.0–5.0)	2.0 (0.0–4.0)	2.0 (0.0–4.0)	0.396
Smoking, *n* (%)	59 (7.7)	22 (8.6)	18 (7.1)	19 (7.5)	0.788
Vital signs					
Temperature, ℃	36.4 (36.4–36.5)	36.6 (36.4–36.9)	57.0 (46.0–72.0)	36.6 (36.1–36.9)	0.054
RR, rpm	20.8 (20.3–21.2)	20.0 (17.5–24.0)	20.0 (16.0–24.0)	20.0 (16.0–24.0)	0.369
HR, rpm	90.4 (88.9–91.8)	88.0 (77.0–106.0)	88.0 (76.8–103.3)	87.0 (78.0–100.0)	0.486
MAP, mmHg	81.5 (80.2–82.8)	77.3 (68.7–88.7)	80.3 (68.5–90.7)	82.0 (70.0–94.7)	0.093
Comorbidity, *n* (%)					
Hypertension	220 (28.8)	50 (19.6)	86 (33.7)	84 (32.9)	0.000
DM	317 (41.4)	129 (50.6)	101 (39.6)	87 (34.1)	0.001
CHF	594 (77.7)	200 (78.4)	200 (78.4)	194 (76.1)	0.763
AF	348 (45.5)	123 (48.2)	124 (48.6)	101 (39.6)	0.069
CVD	98 (12.8)	41 (16.1)	32 (12.5)	25 (9.8)	0.105
COPD	211 (27.6)	71 (27.8)	66 (25.9)	74 (29.0)	0.726
PAD	136 (17.8)	51 (20.0)	47 (18.4)	38 (14.9)	0.304
CKD	279 (36.5)	125 (49.0)	88 (34.5)	66 (25.9)	0.000
Rheumatic disease	37 (4.8)	15 (5.9)	11 (4.3)	11 (4.3)	0.012
Cancer	47 (6.1)	21 (8.2)	14 (5.5)	12 (4.7)	0.219
Severe liver disease	13 (1.7)	7 (2.7)	3 (1.2)	3 (1.2)	0.327
Laboratory findings					
pH	7.3 (7.3–7.3)	7.3 (7.3–7.4)	7.3 (7.3–7.4)	7.3 (7.3–7.4)	0.547
PaCO_2_	41.4 (40.4–42.4)	40.0 (34.0–46.0)	39.0 (34.0–46.0)	41.0 (34.3–48.0)	0.213
PaO_2_	129.8 (121.5–138.1)	89.0 (46.0–169.0)	101.0 (56.0–200.0)	84.0 (53.0–160.8)	0.152
SpO_2_	95.7 (95.3–96.1)	98.0 (93.3–100.0)	97.0 (94.0–100.0)	97.0 (94.0–100.0)	0.890
WBC	14.6 (14.0–15.2)	13.2 (9.1–17.2)	12.1 (8.9–17.0)	13.8 (10.4–19.0)	0.003
RBC	3.8 (3.7–3.8)	3.2 (2.8–3.7)	3.7 (3.3–4.1)	4.3 (3.9–4.8)	0.000
PLT	226.1 (219.1–233.1)	206.0 (150.0–295.0)	207.0 (164.0–261.0)	221.0 (173.0–282.0)	0.159
Potassium	4.5 (4.4–4.5)	4.4 (3.9–4.9)	4.4 (3.9–4.9)	4.3 (3.9–4.8)	0.412
Sodium	137.4 (137.0–137.8)	137.0 (134.0–141.0)	138.0 (135.0–140.0)	138.0 (136.0–141.0)	0.113
Chloride	101.8 (101.3–102.2)	102.0 (96.0–106.0)	103.0 (98.0–106.0)	103.0 (99.0–106.0)	0.129
Bicarbonate	20.6 (20.2–20.9)	20.0 (17.0–23.0)	21.0 (18.0–24.0)	21.0 (18.0–24.0)	0.092
CK-MB	89.1 (79.4–98.9)	20.0 (6.0–61.0)	33.0 (10.0–118.5)	46.0 (11.0–203.0)	0.000
LAC	3.3 (3.1–3.5)	2.1 (1.5–3.9)	2.1 (1.3–3.8)	2.5 (1.7–4.3)	0.052
MV, *n* (%)	477 (62.4)	162 (63.5)	169 (66.3)	146 (57.3)	0.098
IABP, *n* (%)	244 (31.9)	69 (27.1)	86 (33.7)	89 (34.9)	0.122
RRT, *n* (%)	123 (16.1)	55 (21.6)	41 (16.1)	27 (10.6)	0.003
Vasoactive agents, *n* (%)	612 (80.0)	211 (82.7)	210 (82.4)	191 (74.9)	0.045
RBC input	1,958.3 (1,633.1–2,283.6)	793.5 (375.0–1,743.7)	1,400.0 (632.0–3,990.4)	1,000.0 (700.0–2,200.0)	0.010
Plasma input	1,091.8 (823.2–1,360.4)	608.0 (430.0–1,016.3)	625.0 (323.5–1,385.5)	633.0 (459.0–2,265.0)	0.635
Outcomes					
28-day mortality, *n* (%)	341 (44.6)	129 (50.6)	113 (44.3)	99 (38.8)	0.028
90-day mortality, *n* (%)	363 (47.5)	136 (53.3)	121 (47.5)	106 (41.6)	0.029
6-month mortality, *n* (%)	382 (49.9)	144 (56.5)	128 (50.2)	110 (43.1)	0.009
1-year mortality, *n* (%)	407 (53.2)	153 (60.0)	135 (52.9)	119 (46.7)	0.011
LOS-ICU, days	6.1 (5.6–6.6)	4.7 (2.1–9.4)	4.0 (2.1–8.1)	3.2 (1.6–5.2)	0.000
LOS-H, days	13.0 (11.9–14.1)	12.7 (6.8–20.8)	9.9 (5.0–17.8)	6.5 (3.6–11.0)	0.000

### Incidence of all-cause mortality among different groups

**Figure 2:**
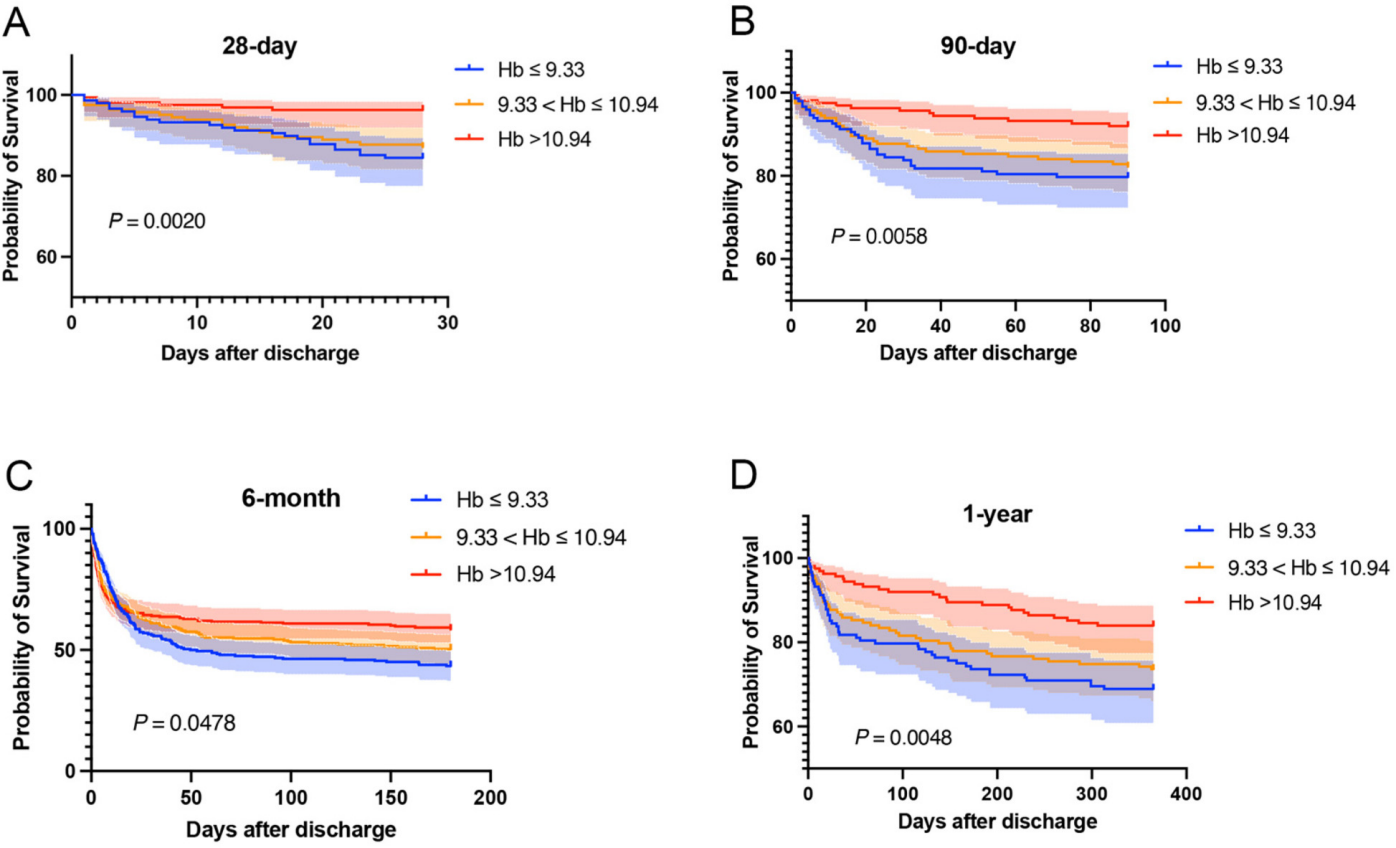
Kaplan–Meier curves regarding 28-day **(A)**, 90-day **(B)**, 6-month **(C)**, and 1-year mortality **(D)** based on time-varying weighted hemoglobin tertiles. Hb, hemoglobin.

**Figure 3:**
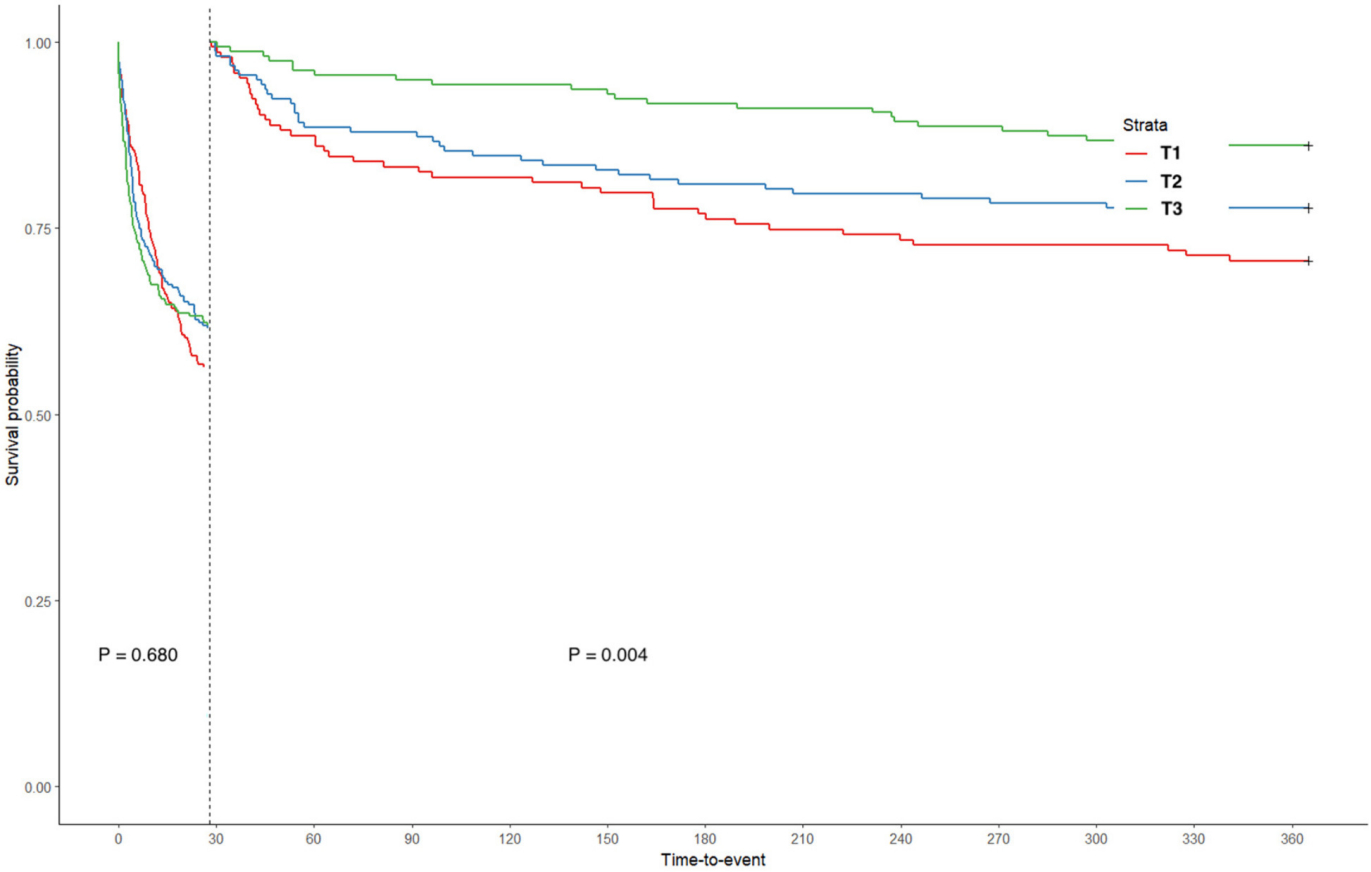
Landmark analysis based on time-varying weighted hemoglobin tertiles. T, tertile.

Kaplan–Meier curves were drawn based on the TWA-Hb tertile, and the analysis showed that the cumulative rates of 28-day, 90-day, 6-month, and 1-year mortality were significantly higher in populations with lower TWA-Hb (log-rank *P* = 0.002, 0.006, 0.048, and 0.005, respectively; Figures 2A–D). Further applying the landmark analyses, findings indicated that, from 28 days to 1 year, all-cause mortality of individuals with the highest TWA-Hb was apparently lower than that of the lowest TWA-Hb, whereas an insignificant result was shown during the short-term follow-up of 28 days (Figure 3). Supplementary Figure 1 depicts the distribution of TWA-Hb stratified according to the survival status of all-cause 28-day, 90-day, 6-month, and 1-year mortality.

### Association between the TWA-Hb and all-cause mortality

Cox regression analyses were further employed to identify the correlation between TWA-Hb and survival events. Statistically increased risks of 90-day (HR = 0.733; 95% CI: 0.569–0.945), 6-month (HR = 0.765; 95% CI: 0.597–0.981), and 1-year (HR = 0.714; 95% CI: 0.562–0.907) mortality, but not 28-day mortality (HR = 0.915; 95% CI: 0.708–1.182), were observed among AMI-CS patients categorized with the lowest TWA-Hb, in comparison with those in the highest category. These results remained significant in the multivariable-adjusted model. Moreover, when TWA-Hb was calculated as a continuous variable, a similar mortality risk tendency was presented at the above time points with decreasing TWA-Hb, after adjusting for confounders (Supplementary Table 2).

### Subgroup analysis

**Figure 4:**
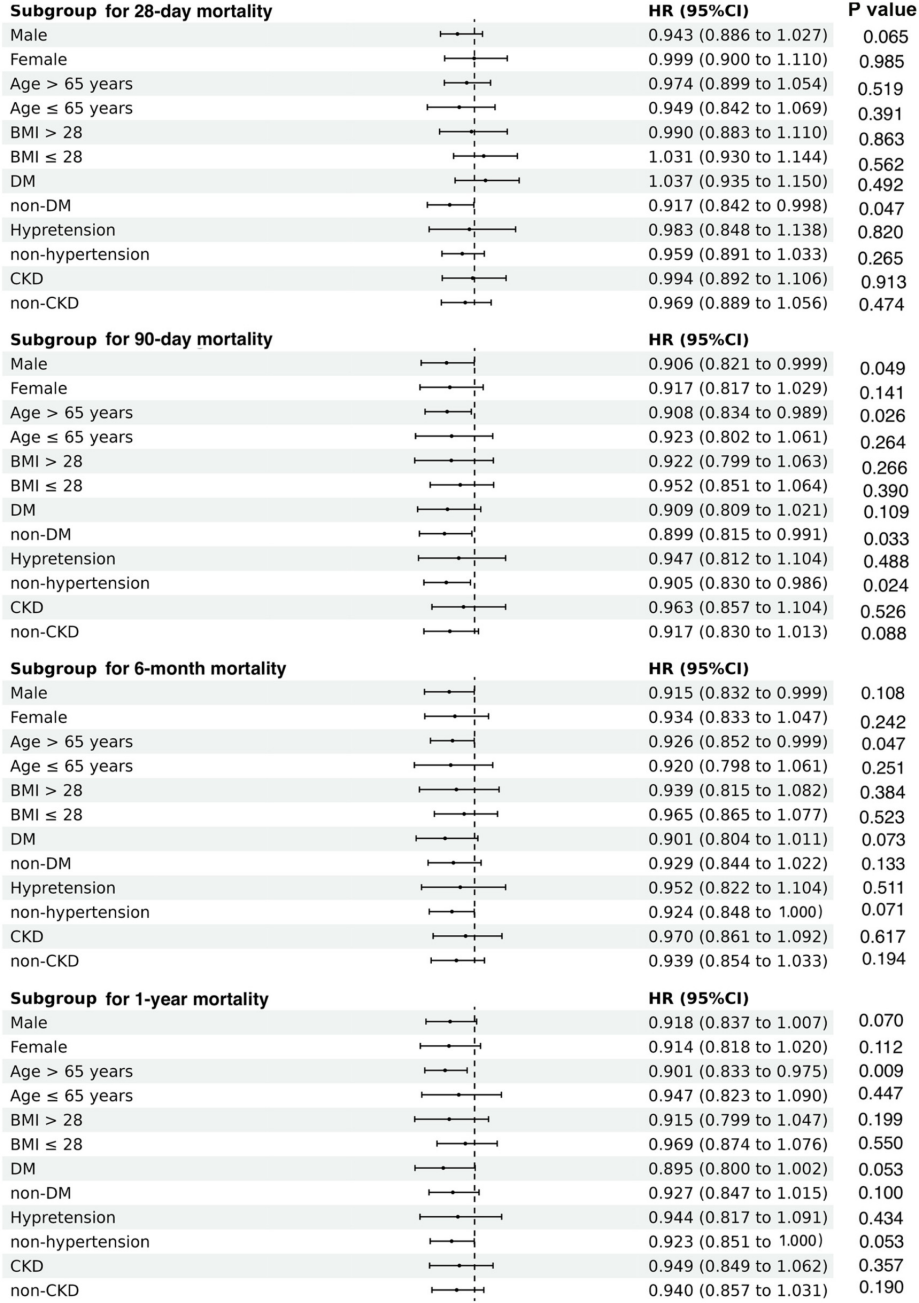
Subgroup analyses regarding the correlation between time-varying weighted hemoglobin and all-cause mortality in patients with acute myocardial infarction complicating cardiogenic shock, according to gender, age, BMI, DM, hypertension, and CKD. BMI, body mass index; DM, diabetes mellitus; CKD, chronic kidney disease.

Subsequently, we performed subgroup analyses to examine the relationship between TWA-Hb levels and all-cause mortality, considering several potential confounding factors such as sex, age, BMI, diabetic status, hypertensive status, and renal function. The results revealed that higher TWA-Hb was apparently correlated with reduced risks of 28-day mortality in the non-DM subgroup (HR = 0.917; 95% CI: 0.842–0.998). Similarly, decreased risks of 90-day mortality were observed in males (HR = 0.906; 95% CI: 0.821–0.999), individuals aged over 65 years (HR = 0.908; 95% CI: 0.834–0.989), those without DM (HR = 0.899; 95% CI: 0.815–0.991), and those without hypertension (HR = 0.905; 95% CI: 0.830–0.986). Furthermore, reduced risks of 6-month and 1-year mortality were noted among those over 65 years (HR = 0.926 and 0.901, respectively; 95% CI: 0.852–0.999 for 6-month and 0.833–0.975 for 1-year) (Figure 4).

## Discussion

Our study demonstrates that lower TWA-Hb levels are significantly associated with elevated long-term mortality in patients with AMI-CS, particularly among elderly individuals and those without DM. To our knowledge, this is the first investigation to establish TWA-Hb—a dynamic metric reflecting cumulative Hb exposure—as an independent prognostic marker in AMI-CS populations.

Existing studies concerning the correlation of Hb with clinical outcomes almost utilized Hb at a single time point and ignored the time-varying characteristic of Hb along with the process of diagnosis and treatment. Notably, anemia is prevalent among AMI-CS patients (16, 17), which might either present on admission or develop during hospitalization as a result of complicated treatments. Moreover, anemia could often trigger RBC transfusions (18). As such, defining Hb status only by using its once-off value could cause potential regression dilution bias and ultimately affect the precision of findings. Lately, an increasing number of studies have investigated the association of cumulative exposure to biological measures (e.g., blood pressure and cholesterol) with the prognosis of cardiovascular disease (19, 20). However, the cumulative effect of Hb on outcomes has not been well studied. A previous study has described that in-hospital Hb drop was apparently correlated with an increased risk for long-term mortality in AMI patients (18). Furthermore, Kalra et al. (21) conducted a multicenter cohort enrolling patients from 45 countries and found that patients with anemia on admission appeared to have no increased mortality risk if their anemia normalized over time. Overall, even during a very short stay in the hospital, the condition evolution of AMI-CS varied rapidly and diversely (1, 22), so the comprehensive cumulative exposure to Hb should be considered when assessing AMI-CS patients' outcomes.

Using the TWA-Hb in the present analysis, we found that lower TWA-Hb presented a strong impact on long-term mortality among patients with AMI-CS, presenting increased mortality at 90-day, 6-month, and 1-year follow-ups. To this day, little is known about the impact of Hb concentrations or anemia presence on the long-term prognosis in patients suffering from AMI-CS. In contrast, concordant with our findings, a study in previous years has linked the improvement of anemia to prolonged survival for non-shock AMI populations (23). Another study also explored that a Hb drop of >3 g/dl during hospitalization was correlated with increased 1-year and 5-year mortality of AMIs (18, 24). Nonetheless, the specific mechanism of lower Hb exposure worsening long-term survival remains inconclusive. One may speculate that inadequate oxygen carriers could impair cellular oxidative metabolism and exaggerate myocardial ischemia, thus promoting the extension of infarcted size (25, 26), as confirmed using SPECT imaging by Dutsch et al. (18) that larger myocardial infarctions were found in those with a Hb decrease of ≥3 g/dl during hospitalization. In the setting of CS, anemia-mediated reduced myocardium oxygen supply would be further jeopardized by CS-associated hypoperfusion, which was more likely to cause a lack of recovery of cardiac pump function after the acute state. In line, a retrospective study including 39,922 STEMI individuals exhibited that baseline Hb <14 g/dl was associated with a higher risk of future heart failure (27). Given that low LVEF has been recognized as one of the pivotal elements related to poor long-term prognosis of cardiovascular diseases (28, 29), we deemed that the reduced myocardial salvage and deteriorated heart function might primarily account for the increased long-term mortality in relation to low levels of TWA-Hb in populations with AMI-CS.

We also evaluated the association between TWA-Hb and short-term mortality (28 days), obtaining insignificant results consistent with those in previous studies regarding STEMI populations but inconsistent with those concerning CS patients. A retrospective study enrolling 1,919 STEMI patients undergoing PCI reported that anemic status did not affect the in-hospital mortality of these patients (30). More recently, another study involving 3,071 STEMI individuals also confirmed that anemia upon admission was not correlated with the risk of in-hospital mortality (31). However, among anemic CS (from any cause) patients, Obradovic et al. (15) found a significantly higher HR for 30-day mortality compared with those without anemia. During this phase, the Hb levels may play a less dominant role in determining immediate survival, as the primary focus is on myocardial reperfusion and circulatory stabilization (32). However, as the patient moves into the sub-acute and chronic phases of recovery, the importance of TWA-Hb and its influence on oxygen delivery to tissues becomes more pronounced. Anemia, even if not immediately life-threatening, can impair oxygen delivery to the myocardium and other organs, potentially impeding recovery and leading to a higher risk of mortality over longer time frames. The chronic reduction in oxygen-carrying capacity can exacerbate myocardial stunning, impair neovascularization, and lead to a vicious cycle of worsening cardiac function and systemic complications. Therefore, while the immediate survival after AMI-CS heavily benefits from timely revascularization and optimal MCS, the longer-term outcomes might be more closely tied to adequate oxygen delivery. Our findings further suggest that TWA-Hb may be a more significant predictor of long-term survival, highlighting the importance of managing anemia in the post-AMI-CS period to improve patient outcomes.

There are still some limitations. First, due to its *post hoc* characteristic, we could not conclude causality between TWA-Hb and all-cause mortality, so further prospective cohorts are required. Second, we could not completely rule out the presence of residual bias, although we performed multi-adjustments for potential confounders. For instance, AMI patients with more severe initial shock were associated with a higher risk of mortality (33). Even after maximal adjustment for confounding factors such as LAC, pH, MAP, the use of vasopressors, and IABP as surrogates for assessing shock severity, the absence of certain data (such as cardiac index and pulmonary capillary wedge pressure) necessary for SCAI classification may have resulted in less accurate risk stratification. Moreover, MCS, such as VA-ECMO and Impella, might hold the potential benefits in improving survival in patients with AMI-CS (7), allowing for myocardial recovery or bridging to decision-making, including the possibility of advanced heart failure therapies. The adoption of these advanced devices might have contributed to better outcomes in patients with AMI-CS, which could have been underrepresented in our initial analysis. Baseline iron status could influence Hb levels and affect the organism's capacity to manage oxidative stress and inflammation, both of which are known to impact prognoses (34). Notably, a history of transfusions might correlate with more severe anemia and other health complications, potentially elevating mortality. Thus, there is a clear need for prospective cohorts with more detailed records. Third, patients enrolled come from a single center, so multicenter studies are necessary to verify the generalizability of these findings. Fourth, data presented in this study, which covers the period from 2008 to 2019, might not reflect the more recent changes in PCI strategies and the broader adoption of advanced MCS devices such as VA-ECMO and Impella. The evolution of PCI techniques, including the use of more sophisticated stenting materials, improved antiplatelet therapies, and enhanced imaging guidance, could potentially improve procedural success rates and reduce complications. Similarly, the increased utilization of MCS devices such as VA-ECMO and Impella might have altered the landscape of AMI-CS treatment, offering new possibilities for patient recovery that are not minutely captured in our study. Therefore, while our study provides valuable historical context, ongoing and future studies with more current data are essential to ensure that clinical guidelines and patient care strategies remain up to date and reflect the latest evidence-based practices. Finally, the function of TWA may not adequately differentiate between patients who experienced a drop in Hb from a high baseline vs. those who had an increase in Hb from a low baseline, preventing a more nuanced analysis of Hb changes in the context of bleeding complications.

## Conclusion

TWA-Hb is an indicator of long-term all-cause mortality among patients suffering from AMI-CS. Serial assessment of TWA-Hb in AMI-CS routine clinical management may improve risk stratification and could thereby guide clinical decision-making. However, additional prospective investigations are still warranted to validate our results.

## Data Availability

The original contributions presented in the study are included in the article/Supplementary Material, further inquiries can be directed to the corresponding authors.
