# Supplementary material for: Association between time-varying weighted hemoglobin and all-cause mortality in patients with acute myocardial infarction-related cardiogenic shock
Source: Front Cardiovasc Med. 2025 May 14;12:1516100. doi: 10.3389/fcvm.2025.1516100 (PMC12116649; doi:10.3389/fcvm.2025.1516100)
Supplement: Supplementary file 1 [file Table1.docx]

**Supplementary Table 1. Univariate logistic regression analysis.**

| Variables | OR (95% CI) | *P* value |
| --- | --- | --- |
| Hb, g/L  Age, years  Gender  BMI, kg/m^2^  Ethnicity  Smoking  Temperature, ℃  RR, rpm  HR, rpm  MAP, mmHg  Hypertension  DM  CHF  AF  CVD  COPD  PAD  CKD  Rheumatic disease  Cancer  Severe liver disease  pH  PaCO2  PaO2  SpO2  WBC  RBC  PLT  Potassium  Sodium  Chloride  Bicarbonate  CK-MB  LAC  MV  IABP  RRT  Vasoactive agents  RBC input  Plasma input | 0.880 (0.811-0.956)  1.038 (1.025-1.051)  3.313 (2.450-4.481)  0.990 (0.966-1.014)  1.259 (1.075-1.475)  1.314 (0.772-2.236)  0.697 (0.587-0.826)  1.045 (1.020-1.070)  1.005 (0.998-1.012)  0.988 (0.980-0.996)  0.656 (0.476-0.904)  1.377 (1.031-1.840)  0.679 (0.483-0.955)  1.063 (0.799-1.416)  1.169 (0.765-1.788)  1.053 (0.766-1.448)  1.401 (0.966-2.031)  2.162 (1.602-2.917)  0.945 (0.485-1.840)  2.097 (1.143-3.844)  1.460 (0.486-4.386)  0.022 (0.005-0.091)  1.002 (0.990-1.015)  1.000 (0.998-1.001)  0.965 (0.940-0.991)  1.018 (0.999-1.037)  0.669 (0.558-0.801)  0.999 (0.997-1.000)  1.104 (0.934-1.306)  1.020 (0.993-1.047)  0.989 (0.969-1.010)  0.893 (0.863-0.923)  0.999 (0.997-1.000)  1.341 (1.247-1.443)  1.592 (1.181-2.145)  0.787 (0.578-1.071)  2.604 (1.744-3.888)  1.738 (1.200-2.517)  1.000 (1.000-1.000)  1.001 (1.000-1.001) | 0.002  < 0.001  < 0.001  0.391  0.004  0.314  < 0.001  < 0.001  0.174  0.005  0.010  0.030  0.026  0.674  0.471  0.751  0.075  < 0.001  0.867  0.017  0.500  < 0.001  0.075  0.638  0.009  0.059  < 0.001  0.058  0.248  0.150  0.318  < 0.001  0.072  < 0.001  0.002  0.128  < 0.001  0.003  0.041  0.068 |
