# Supplementary material for: Association between time-varying weighted hemoglobin and all-cause mortality in patients with acute myocardial infarction-related cardiogenic shock
Source: Front Cardiovasc Med. 2025 May 14;12:1516100. doi: 10.3389/fcvm.2025.1516100 (PMC12116649; doi:10.3389/fcvm.2025.1516100)
Supplement: Supplementary file 2 [file Table2.docx]

**Supplementary Table 2.** Cox proportional HRs for all-cause mortality.

| Variables | Model 1  HR (95%CI) | P value | Model 2  HR (95%CI) | P value | Model 3  HR (95%CI) | P value |
| --- | --- | --- | --- | --- | --- | --- |
| 28-day mortality  Per unit increase  T1 (n = 255)  T2 (n = 255)  T3 (n = 255) | 0.950 (0.897-1.005)  1 (Reference)  1.328 (1.048-1.683)  0.915 (0.708-1.182) | 0.075  -  0.019  0.495 | 0.950 (0.897-1.006)  1 (Reference)  1.285 (1.011-1.631)  0.905 (0.700-1.171) | 0.081  -  0.040  0.448 | 0.950 (0.830 ~ 1.088)  1 (Reference)  1.092 (0.808-1.476)  1.274 (0.932-1.741) | 0.463  -  0.568  0.129 |
| 90-day mortality  Per unit increase  T1 (n = 255)  T2 (n = 255)  T3 (n = 255) | 0.914 (0.859-0.971)  1 (Reference)  0.844 (0.661-1.078)  0.733 (0.569-0.945) | 0.004  -  0.175  0.017 | 0.915 (0.858-0.976)  1 (Reference)  0.753 (0.585-0.968)  0.726 (0.561-0.939) | 0.007  -  0.027  0.015 | 0.933 (0.878-0.992)  1 (Reference)  1.161 (0.905-1.489)  0.746 (0.584-0.952) | 0.026  -  0.240  0.019 |
| 6-month mortality  Per unit increase  T1 (n = 255)  T2 (n = 255)  T3 (n = 255) | 0.925 (0.871-0.982)  1 (Reference)  0.873 (0.688-1.108)  0.765 (0.597-0.981) | 0.010  -  0.265  0.035 | 0.928 (0.872-0.988)  1 (Reference)  0.775 (0.607-0.989)  0.760 (0.591-0.978) | 0.020  -  0.040  0.033 | 0.758 (0.596 ~ 0.962)  1 (Reference)  1.041 (0.838-1.293)  0.704 (0.545-0.910) | 0.023  -  0.717  0.007 |
| 1-year mortality  Per unit increase  T1 (n = 255)  T2 (n = 255)  T3 (n = 255) | 0.909 (0.858-0.963)  1 (Reference)  0.827 (0.656-1.043)  0.714 (0.562-0.907) | 0.001  -  0.109  0.006 | 0.928 (0.874-0.985)  1 (Reference)  0.795 (0.630-1.004)  0.771 (0.605-0.981) | 0.014  -  0.054  0.035 | 0.706 (0.526-0.948)  1 (Reference)  0.839 (0.585-1.204)  0.705 (0.505-0.984) | 0.021  -  0.341  0.040 |

Model 1: unadjusted.

Model 2: adjusted for age and gender.

Model 3: adjusted for age, gender, BMI, ethnicity, temperature, respiratory rate, mean arterial pressure, hypertension, diabetes mellitus, chronic heart failure, chronic kidney disease, cancer, pH, SpO2, red blood cell, bicarbonate, lactate, intra-aortic ballon pump use, mechanical ventilation use, renal replacement therapy, vasoactive agents use, and RBC input.
