# Supplementary figures and images for: Association between time-varying weighted hemoglobin and all-cause mortality in patients with acute myocardial infarction-related cardiogenic shock
Source: Front Cardiovasc Med. 2025 May 14;12:1516100. doi: 10.3389/fcvm.2025.1516100 (PMC12116649; doi:10.3389/fcvm.2025.1516100)

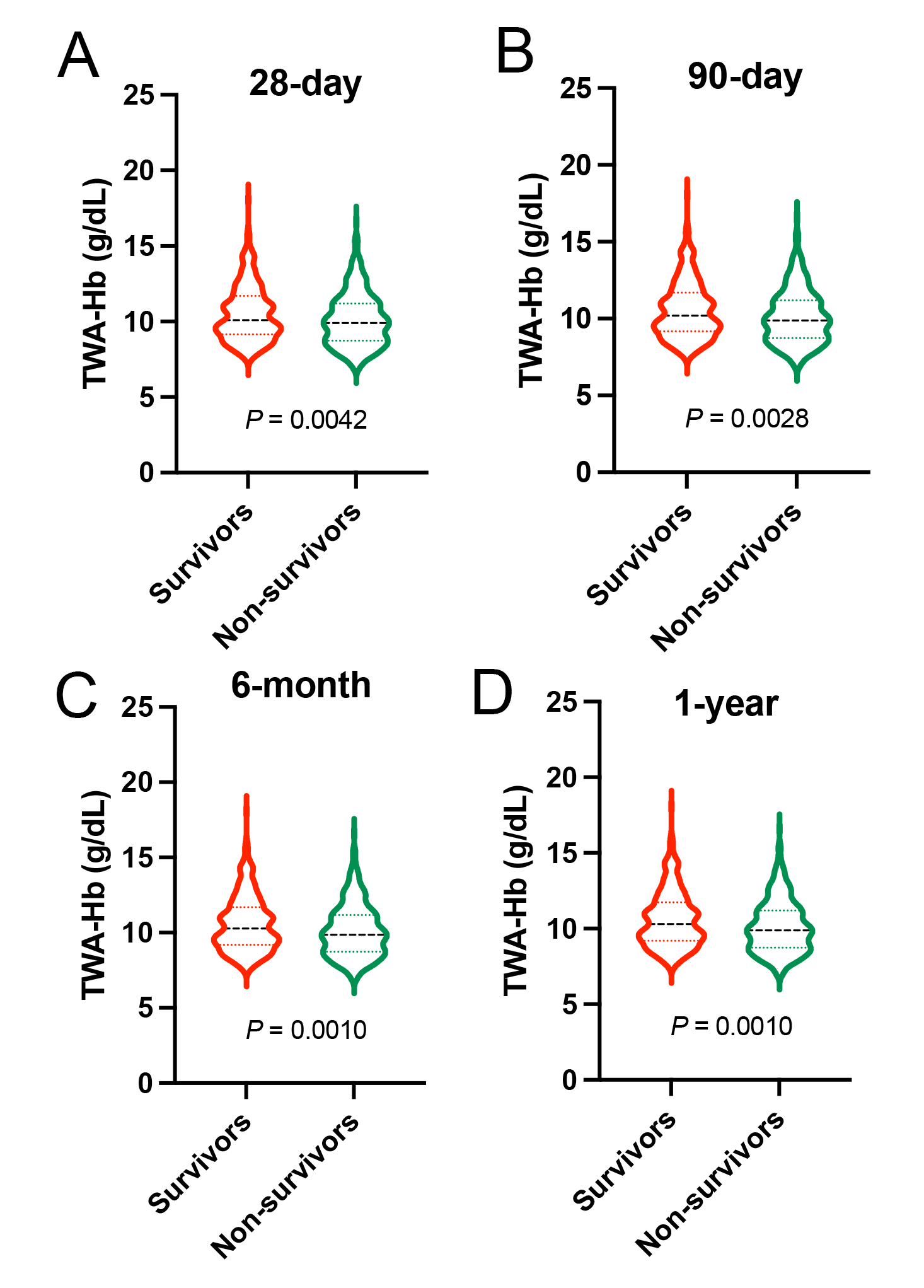

Supplement: Supplementary file 3 [file Image1.tif]
